# A novel TBK1/IKKϵ is involved in immune response and interacts with MyD88 and MAVS in the scallop *Chlamys farreri*

**DOI:** 10.3389/fimmu.2022.1091419

**Published:** 2023-01-12

**Authors:** Wenjuan Liu, Jilv Ma, Jiwen Chen, Baoyu Huang, Fengchen Liu, Lingling Li, Nini Fan, Fangshu Li, Yanxin Zheng, Xuekai Zhang, Xiaona Wang, Xiaomei Wang, Lei Wei, Yaqiong Liu, Meiwei Zhang, Yijing Han, Xiaotong Wang

**Affiliations:** ^1^ School of Agriculture, Ludong University, Yantai, China; ^2^ Ocean School, Yantai University, Yantai, China; ^3^ Changdao Enhancement and Experiment Station, Chinese Academy of Fishery Sciences, Yantai, China

**Keywords:** *Chlamys farreri*, innate immunity, TBK1/IKKϵ, MyD88, MAVS

## Abstract

Inhibitor of κB kinase (IKK) family proteins are key signaling molecules in the animal innate immune system and are considered master regulators of inflammation and innate immunity that act by controlling the activation of transcription factors such as NF-κB. However, few functional studies on IKK in invertebrates have been conducted, especially in marine mollusks. In this study, we cloned the IKK gene in the Zhikong scallop *Chlamys farreri* and named it *CfIKK3*. *CfIKK3* encodes a 773-amino acid-long protein, and phylogenetic analysis showed that CfIKK3 belongs to the invertebrate TBK1/IKKϵ protein family. Quantitative real-time PCR analysis showed that *CfIKK3* mRNA is ubiquitously expressed in all tested scallop tissues. The expression of *CfIKK3* transcripts was significantly induced after challenge with lipopolysaccharide, peptidoglycan, or poly(I:C). Co-immunoprecipitation (co-IP) assays confirmed the direct interaction of CfIKK3 with MyD88 (the key adaptor in the TLR pathway) and MAVS (the key adaptor in the RLR pathway), suggesting that this IKK protein plays a crucial role in scallop innate immune signal transduction. In addition, the CfIKK3 protein formed homodimers and bound to CfIKK2, which may be a key step in the activation of its own and downstream transcription factors. Finally, in HEK293T cells, dual-luciferase reporter gene experiments showed that overexpression of CfIKK3 protein activated the NF-κB reporter gene in a dose-dependent manner. In conclusion, our experimental results confirmed that CfIKK3 could respond to PAMPs challenge and participate in scallop TLR and RLR pathway signaling, ultimately activating NF-κB. Therefore, as a key signaling molecule and modulator of immune activity, CfIKK3 plays an important role in the innate immune system of scallops.

## 1 Introduction

Animals live in an environment rich in pathogenic microorganisms such as viruses and bacteria, and in the long evolutionary process, they have evolved a perfect immune system to fight the invasion of these pathogens. The immune system includes the innate immune system as well as the acquired immune system. It is generally believed that only vertebrates have a remarkably well-developed adaptive immune system, whereas invertebrates usually rely on the innate immune system to fight the invasion of pathogenic microorganisms (1). Innate immunity begins with the recognition of pathogen-associated molecular patterns (PAMPs) by pattern recognition receptors (PRRs). Common PRRs include RIG-I-like receptors (RLRs), toll-like receptors (TLRs), and NOD-like receptors (NLRs), amongst others (2). After pathogen recognition by PRRs, many signal transduction molecules need to be recruited for the transmission of immune signals, and finally for the activation of some key transcription factors such as interferon regulatory factors (IRFs) and nuclear factor-kappa B (NF-κB). These transcription factors further enter the nucleus to regulate downstream gene expression, ultimately killing the invading pathogens (3, 4).

Inhibitors of NF-κB (IκB) kinases (IKKs) are pleiotropic signaling molecules involved in innate immune signal transduction, including the classical IKK genes *IKKα* and *IKKβ* and the IKK-related genes TANK-binding kinase 1 (*TBK1*) and *IKKϵ* (5). IKKα and IKKβ along with another scaffold protein known as IKKγ or NF-κB essential modulator (NEMO) form the canonical IKK complex, which plays an important role in IκB phosphorylation and activation of the transcription factor NF-κB (6, 7). The IKK-related kinases TBK1 and IKKϵ are also known for their critical roles in innate immunity (5). *TBK1* and *IKKϵ*, originally identified as homologs of *IKKα* and *IKKβ*, possess the ability to activate NF-κB at the same time (8, 9). However, the ability of *TBK1* and *IKKϵ* to activate *IRF* is more established (10). In vertebrates, TBK1 and IKKϵ generally interact with TRAF (TNF receptor-associated factor) family proteins (such as TRAF3) to receive upstream immune signals (11). TBK1 and IKKϵ phosphorylate IRF3 and IRF7, both of which homodimerize and translocate into the nucleus, where they form enhancer complexes with NF-κB and other transcription factors to initiate the transcription of type I interferon genes (12).

Compared with the innate immune system of vertebrates, the innate immunity of invertebrates, especially of marine mollusks, are poorly understood particularly with respect to the signal transduction of innate immune pathways. The *Drosophila melanogaster* genome encodes the homologous genes of the IKK family, namely immune response deficient 5 (*IRD5*) and *Kenny*, which are the homologous genes of vertebrate *IKKβ* and *IKKγ*, respectively (13, 14). *IRD5* and *Kenny* binds to each other to form a signaling complex, which can mediate the *Drosophila* immune deficiency pathway and activate antibacterial gene expression (15). Sporadic reports on the identification and functional validation of *IKK* genes of other invertebrates are available. For example, IKK of disk abalone responds to bacterial and viral stimuli and activates the *NF-κB* reporter gene in mammalian cells (16). The IKKα/IKKβ proteins from the Pacific oyster seem to be a group of multifunctional immune signaling molecules that can play a key role in host antiviral and bacterial immunity by participating in the signal transduction of oyster TLR and RLR innate immune pathways (17, 18). Recent studies have found that scallop IKK1 can also participate in the innate immune process of host cells by interacting with the key adaptor molecule MyD88 (myeloid differentiation primary response gene 88) in the TLR pathway (19). These studies preliminarily revealed that invertebrate IKKs could respond to pathogen stimuli and their PAMPs and participate in the host’s innate immune signal transduction process, which is helpful for dissecting invertebrate innate immune mechanisms. On the one hand, the details of invertebrate IKK-mediated signal transduction and immune function are largely unknown. On the other hand, there may also be a significant number of *IKK* genes expanded in marine mollusks (20, 21); therefore, the study of the functions of these IKKs is particularly interesting.

Many marine mollusks are economically important species for farming; they are delicious, nutritious, and a high-quality protein source. The Zhikong scallop is an economically important aquaculture mollusk distributed in the coastal areas of China, Japan, and South Korea (22). In China, this organism is bred at a large scale and its output value is high. However, in the past two decades, large-scale mortality due to disease has caused huge losses to the scallop farming industry (23, 24). Therefore, it is very important to conduct in-depth research on the key immune genes of scallops to provide necessary theoretical support for the cultivation of disease-resistant strains.

In view of the possible key functions of IKK in innate immunity and the lack of related research on mollusks, we performed sequence identification and functional analysis of the IKK gene in Zhikong scallops. We detected the expression patterns of *CfIKK3* mRNA under attack by different PAMPs and studied the ability of CfIKK3-mediated immune signal transduction to activate key transcription factors. Our findings are beneficial for the analysis of invertebrate innate immune mechanisms and lay a foundation for the development of comparative immunology and formulation of future disease resistance strategies in scallops.

## 2 Materials and methods

### 2.1 Animals

The Zhikong scallops (*Chlamys farreri*) used in this study were collected from a local farm in Yantai, Shandong province, China. The studies involving animals were reviewed and approved by the respective Animal Research and Ethics Committees of Ludong University. The animals had shell height of 52–58 mm. The scallops were cultured in filtered and aerated seawater at 19 ± 1°C for one week before processing.

### 2.2 cDNA sequence confirmation and analysis

The genome of the Zhikong scallop has been sequenced (25), and based on *IKK* gene sequence alignment, we found several *IKK* homologous genes in the *C*. *farreri* genome. In this study, we focused on one *IKK* gene, and the forward and reverse primers, CfIKK3-F and CfIKK3-R, respectively, were designed for PCR amplification of its open reading frame (ORF) (**Table 1**). The PCR products were purified using a SanPrep column DNA gel extraction kit (Sangon Biotech, Shanghai, China) and ligated into the pEASY-T1 cloning vector (TransGen Biotech, Beijing, China). Positive colony clones were sequenced to obtain the complete coding sequence for this gene. The coding amino acid sequences were obtained using an online translation tool (http://web.expasy.org/translate/). The CDD/SPARCLE database (https://www.ncbi.nlm.nih.gov/Structure/cdd/wrpsb.cgi) and simple modular architecture research tool (SMART, http://smart.embl-heidelberg.de/) were employed to predict the protein domains of CfIKK3. The IKK family protein sequences of the different species were acquired from the NCBI database (http://www.ncbi.nlm.nih.gov/guide/proteins/). Multiple sequence alignments were performed using Clustal Omega (https://www.ebi.ac.uk/Tools/msa/clustalo/). Phylogenetic analysis was performed based on the IKK protein phylogenetic tree, which was constructed using the neighbor-joining algorithm in MEGA software (v.5.05) (http://www.megasoftware.net).

**Table 1:** Primers used in this research.

Primers	Sequence(5’-3’)	Application
CfIKK3-F	ATGGCTCAATCTACACTGAGAG	ORF cloning
CfIKK3-R	TCATCCAAATCCGGAGGTCTTTTC	ORF cloning
CfIIKK3-qRT-F	ACGGCATTGATAGGGATACG	qRT-PCR
CfIKK3-qRT-R	CATTCAGAAGCGGAGGGTTT	qRT-PCR
CfEF-1α-QF	GCCATACCGCTCACATTGCT	qRT-PCR
CfEF-1α-QR	CCAGAACGACGGTCGAGTTT	qRT-PCR
CfMyD88-Myc-F	CATGGAGGCCCGAATTATGGCAATGGCGGATATCGA	Protein expression
CfMyD88-Myc-R	CTCGGTCGACCGAATTTTACTCTCCTCGTTTTTTATTTTT	Protein expression
CfMAVS-6Myc-F	CAGTGGCGGCCGCTCGATGAAGATGGCGTTTATAAACGAT	Protein expression
CfMAVS-6Myc-R	GGGCCCTCTAGACTCGATTATTTTGTTTTAGCCAAAGCTATG	Protein expression
CfIKK3-FL-Myc-F	CATGGAGGCCCGAATTATGGCTCAATCTACACTGAGAG	Protein expression
CfIKK3-FL-Myc-R	CTCGGTCGACCGAATTTCATCCAAATCCGGAGGTCTTTTC	Protein expression
CfIKK3-P1-Myc-R	CTCGGTCGACCGAATTTCCAAAGGATAGTTTATACCTG	Protein expression
CfIKK3-P3-Myc-F	CATGGAGGCCCGAATTAAATCGTGGTGTCCAGACTTCCC	Protein expression
CfIKK3-FL-FLAG-F	CTCCATATGACTAGTCTCGAGATGGCTCAATCTACACTGAGAG	Protein expression
CfIKK3-FL-FLAG-R	TACCACGCGTGAATTCTCGAGTCATCCAAATCCGGAGGTCTTTTC	Protein expression
CfIKK3-P1-FLAG-R	TACCACGCGTGAATTCTCGAGTCCAAAGGATAGTTTATACCTG	Protein expression
CfIKK3-P2-FLAG-R	TACCACGCGTGAATTCTCGAGGGGAAAAGTCCTCTGGTCATC	Protein expression
CfIKK3-P3-FLAG-F	CTCCATATGACTAGTCTCGAGAAATCGTGGTGTCCAGACTTCCC	Protein expression
CfMyD88-FL-FLAG-F	CTCCATATGACTAGTCTCGAGATGGCAATGGCGGATATCGA	Protein expression
CfMyD88-FL-FLAG-R	TACCACGCGTGAATTCTCGAGTTACTCTCCTCGTTTTTTATTTTT	Protein expression
CfMyD88-P1-FLAG-R	TACCACGCGTGAATTCTCGAGTTCTTCCAATTGAGGAGATCGA	Protein expression
CfMyD88-P2-FLAG-R	TACCACGCGTGAATTCTCGAGGTCGTAATATATCTCATCTTTAC	Protein expression
CfMyD88-P3-FLAG-R	TACCACGCGTGAATTCTCGAGCACAAAATTCAATATACTAGGAGG	Protein expression
CfMAVS-FL-FLAG-F	CTCCATATGACTAGTCTCGAGATGAAGATGGCGTTTATAAACGAT	Protein expression
CfMAVS-FL-FLAG-R	TACCACGCGTGAATTCTCGAGTTATTTTGTTTTAGCCAAAGCTATG	Protein expression
CfMAVS-P1-FLAG-R	TACCACGCGTGAATTCTCGAGGTACTTTCTTGATCTGGAAGAC	Protein expression
CfMAVS-P2-FLAG-R	TACCACGCGTGAATTCTCGAGCTGGTAACCAGTAACCTTTTGC	Protein expression
CfMAVS-P3-FLAG-R	TACCACGCGTGAATTCTCGAGGGTTTTAGGGTATGACAATGCA	Protein expression

### 2.3 Immune stimulation and sample collection

After acclimation, the 160 scallops were randomly divided into four groups of 40 scallops each. Each group of scallop was injected intramuscularly with any of 50 μL phosphate-buffered saline (PBS; 0.14 M NaCl, 3 mM KCl, 10 mM Na_2_HPO_4_, and 1.5 mM KH_2_PO_4_, pH 7.4), 50 μL lipopolysaccharide (LPS; 10 mg/mL in PBS; Sigma, USA), 50 μL peptidoglycan (PGN; 10 mg/mL in PBS; Sigma), or 50 μL polyinosinic–polycytidylic acid (poly(I:C); 1.0 mg/mL in PBS; *In vivo*Gen, USA). The gills of three animals from each group were randomly sampled at 0, 3, 6, 12, 24, 48, and 72 h after PBS, LPS, PGN, and poly(I:C) stimulation. In addition, nine types of tissues (gills, hemocytes, feet, striated muscle, smooth muscle, hepatopancreas, gonad, mantle, and kidney) were collected from three untreated scallops to study the tissue distribution of *CfIKK3* mRNA.

### 2.4 *CfIKK3* mRNA expression profiles analysis

A Tiangen RNA extraction kit (Tiangen, China) was used to isolate total RNA from the scallop tissues. Reverse transcription PCR was performed using a PrimeScript RT reagent kit with gDNA eraser (Takara, Japan). qRT-PCR was performed using SYBR Green 2× Master Mix (Takara) and an ABI 7500 real-time thermal cycler (Applied Biosystems, USA). Primers CfIKK3-qRT-F/R were used to amplify the target gene fragments; qRT-PCR was programmed as follows: 6 min at 95°C, followed by 40 cycles of 10 s at 95°C and 30 s at 60°C. The specificity of the PCR products was analyzed using a melting curve. Relative gene expression levels were evaluated using the 2^–ΔΔCt^ method (26). The housekeeping *EF-1α* gene (GenBank accession number DT716075.1) was used as the internal control gene. The *CfIKK3* expression levels following PAMP challenge were normalized to the expression in PBS-treated scallops. Differences were considered significant if the *p-*value was less than 0.05.

### 2.5 Cell culture, plasmids construction, and transfection

Currently, no mollusk cell line can successfully transfect protein-expression plasmids. Human embryonic kidney (HEK) 293T cells were used for protein expression. The cell lines present in this study were obtained from the American Type Culture Collection. Cells were grown in Dulbecco’s modified Eagle’s medium (Sigma, USA) supplemented with 10% fetal bovine serum (Gibco, USA) and 1× penicillin–streptomycin solution (Sangon, China). HEK293T cells were grown at 37°C in 5% CO_2_ and subcultured every 2–3 days.

For the expression of Myc- and FLAG-tagged proteins, PCR fragments encoding full-length or truncated mutants were amplified using Phusion High-Fidelity DNA polymerase (Thermo Fisher Scientific, USA). Gene-specific primers used for amplification are listed in **Table 1**. For Myc-tagged protein expression, plasmids pCMV-Myc (Clontech, USA) or pcDNA3.0-6×Myc (constructed in our lab, based on pcDNA3.0) were linearized with *EcoR*I (New England Biolabs, USA) or *Xho*I (New England Biolabs), respectively. For FLAG-tagged protein expression, the plasmid pCMS-EGFP-FLAG (constructed in our laboratory, based on pCMS-EGFP) was digested with *Xho*I (New England Biolabs). The purified PCR products were then inserted into the digested plasmid using an OK Clon DNA ligation kit (Accurate Biotechnology (Hunan) Co., Ltd., China).

The constructed protein expression plasmid was transfected into HEK293T cells using lipofectamine 3000 reagent (Invitrogen) according to the manufacturer’s instructions.

### 2.6 Co-immunoprecipitation assay

HEK293T cells were grown to an appropriate density in 10 cm dishes (Corning, USA). Afterwards, the cells were co-transfected with 5 μg Myc-tagged protein expression plasmid and 5 μg FLAG-tagged plasmid (pCMS-FLAG as a control). Twenty-four hours after transfection, the cells were harvested and lysed using a cell lysis buffer (Beyotime, China). Next, 20 μL of cell lysate was isolated as an input sample to detect protein expression. An appropriate amount of anti-FLAG M2 magnetic beads (Sigma-Aldrich) was added to the remaining lysate samples. The mixture was incubated at 4°C for > 2 h with gentle shaking, after which the beads were rinsed 3 times with cell lysis buffer. Twenty microliters of input samples and rinsed magnetic beads were then mixed with 2× protein sodium dodecyl sulfate-polyacrylamide gel electrophoresis loading buffer (Takara) at 100°C under denaturation for 5 min. Western blot assays were employed for target protein examination using an anti-Myc antibody (Abbkine Scientific Co., Ltd., Wuhan, China) and an anti-FLAG antibody (Sigma).

### 2.7 Western blot assays

Proteins were extracted from HEK293T cells using RIPA lysis buffer (Beyotime, China), separated on a 10% SDS-PAGE gel, and transferred to PVDF membranes (Roche). After blocking with 3% nonfat milk in TBST (10 mM Tris-HCl, pH 7.4, 150 mM NaCl, and 0.05% Tween-20) for 2 h, the membrane was incubated with a primary antibody (anti-Myc or FLAG antibody in TBST; 1:1000 dilution) for 3 h. After three washes in TBST, horseradish peroxidase-labeled goat anti-mouse IgG (Abbkine Scientific Co., Ltd., diluted 1:1000 in TBST) was used as the secondary antibody and incubated for 2 h at room temperature. Unbound IgG was then washed away, and the signals were visualized with Meilunbio^®^ fg super sensitive ECL luminescence reagent (Meilun Biotechnology Co., Ltd., Dalian, China), according to the manufacturer’s instructions. Protein bands were obtained using a ProteinSimple FluorChem E ultrasensitive, fully automated imaging analysis system (Bio-Techne, USA).

### 2.8 Dual-luciferase reporter gene assays

HEK293T cells were used to overexpress the CfIKK3 protein in order to study the activation of reporter genes such as NF-κB and interferon-stimulated response element (ISRE) using dual-luciferase reporter (DLR) experiments. HEK293T cells were cultured in 24-well plates and grown to approximately 50% density. The cells were then co-transfected with CfIKK3 (or its truncated expression plasmid), NF-κB reporter plasmid or ISRE reporter plasmid (Beyotime, China), and Renilla pRL-TK internal control vector (Promega, USA). Cells were harvested 24 h after transfection, and the transcriptional activity of the reporter genes was assayed using the Firefly & Renillalight luciferase reporter assay kit (Meilun Biotechnology Co., Ltd., Dalian, China). Firefly luciferase activity was normalized to Renilla luciferase activity. To ensure reliability, experiments in each group were performed in triplicate.

## 3 Results

### 3.1 Gene cloning

**Figure 1:**
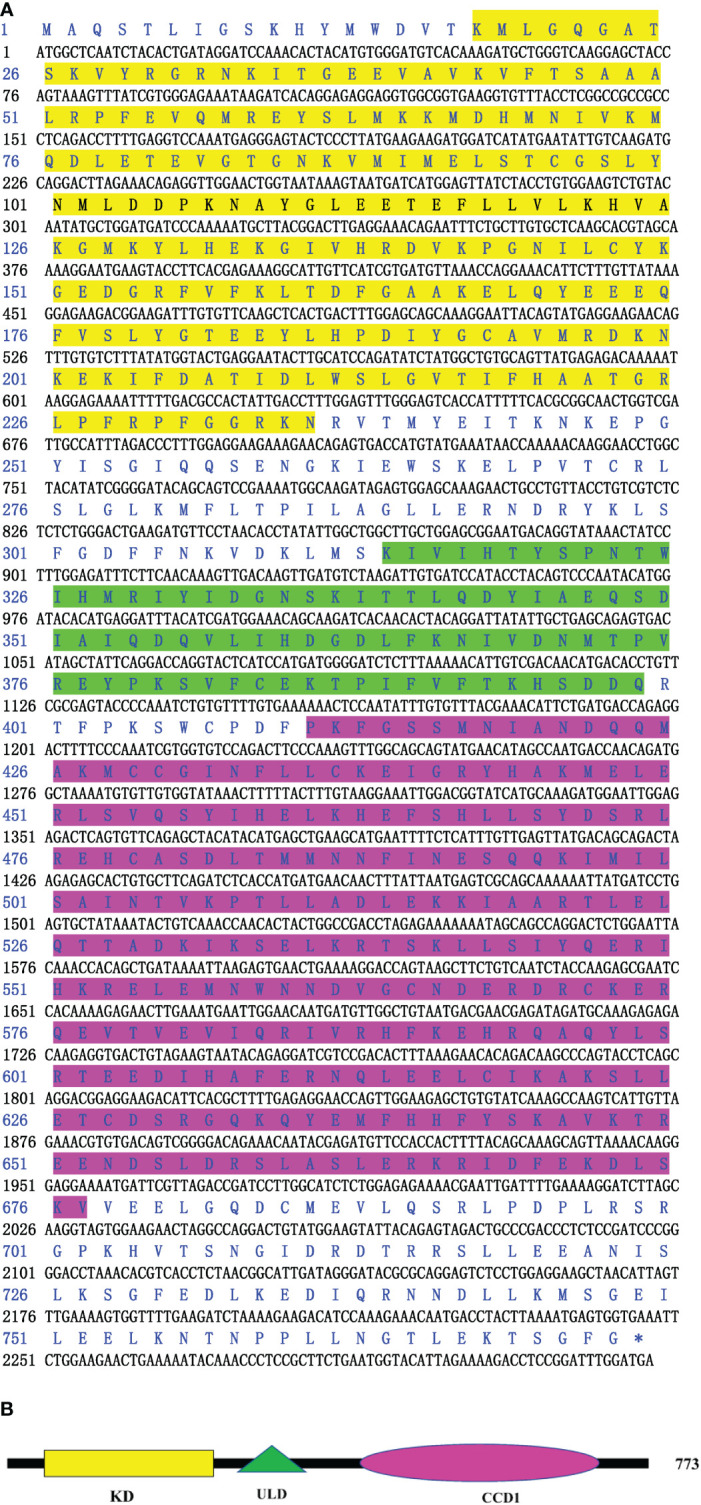
Nucleotide and deduced amino acid sequence of *CfIKK3* **(A)** and schematic representation of CfIKK3 protein **(B)**. The KD (highlighted in yellow on the sequence), ULD (green highlighted), and CDD1 (fuchsia highlighted) were predicted using the NCBI Conserved Domain search program (https://www.ncbi.nlm.nih.gov/Structure/cdd/wrpsb.cgi) and Simple Modular Architecture Research Tool (SMART, http://smart.embl-heidelberg.de/). KD, kinase domain; ULD, ubiquitin-like domain; CCD1, coiled-coil domain 1.

Based on the *C. farreri* genome (25), the primers CfIKK3-F and CfIKK3-R were designed to amplify the ORF of the scallop *IKK* gene. The complete coding sequence was confirmed and submitted to the GenBank database (GenBank accession number OP764589). Because this is the third IKK gene sequence we obtained from the *C. farreri* scallop genome, we named it *CfIKK3*. The polypeptide encoded by CfIKK3 is 773 amino acids long. The N-terminus of CfIKK3 protein is characterized by a typical protein kinase domain like that of other IKK family proteins. Two more domains, namely, a ubiquitin-like domain (ULD) and coiled-coil domain 1 (CCD1) were also present in IKK3 (**Figure 1**).

### 3.2 Sequence analysis

**Figure 2:**
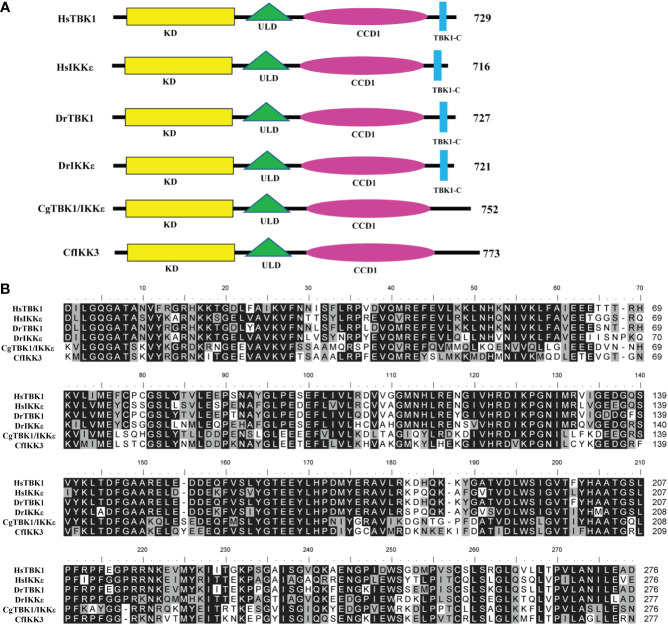
Schematic representation of TBK1/IKKϵ proteins of different species **(A)** and multiple sequence alignment of the kinase domain of TBK1/IKKϵ proteins from different species **(B)**. The GenBank accession numbers for the sequences used are as follows: HsTBK1, NP_037386.1; HsIKKϵ, NP_054721.1; DrTBK1, NP_001038213.2; DrIKKϵ, NP_001002751.1; CgTBK1/IKKϵ, XP_034304979.1. Hs, *Homo sapiens*; Dr, *Danio rerio*; Cg, *Crassostrea gigas*; Cf, *Chlamys farreri*. KD, kinase domain; ULD, ubiquitin-like domain; CCD1, coiled-coil domain 1. Sequences with identical amino acids are shaded in black, whereas conservative amino acid substitutions are shaded in gray.

**Figure 3:**
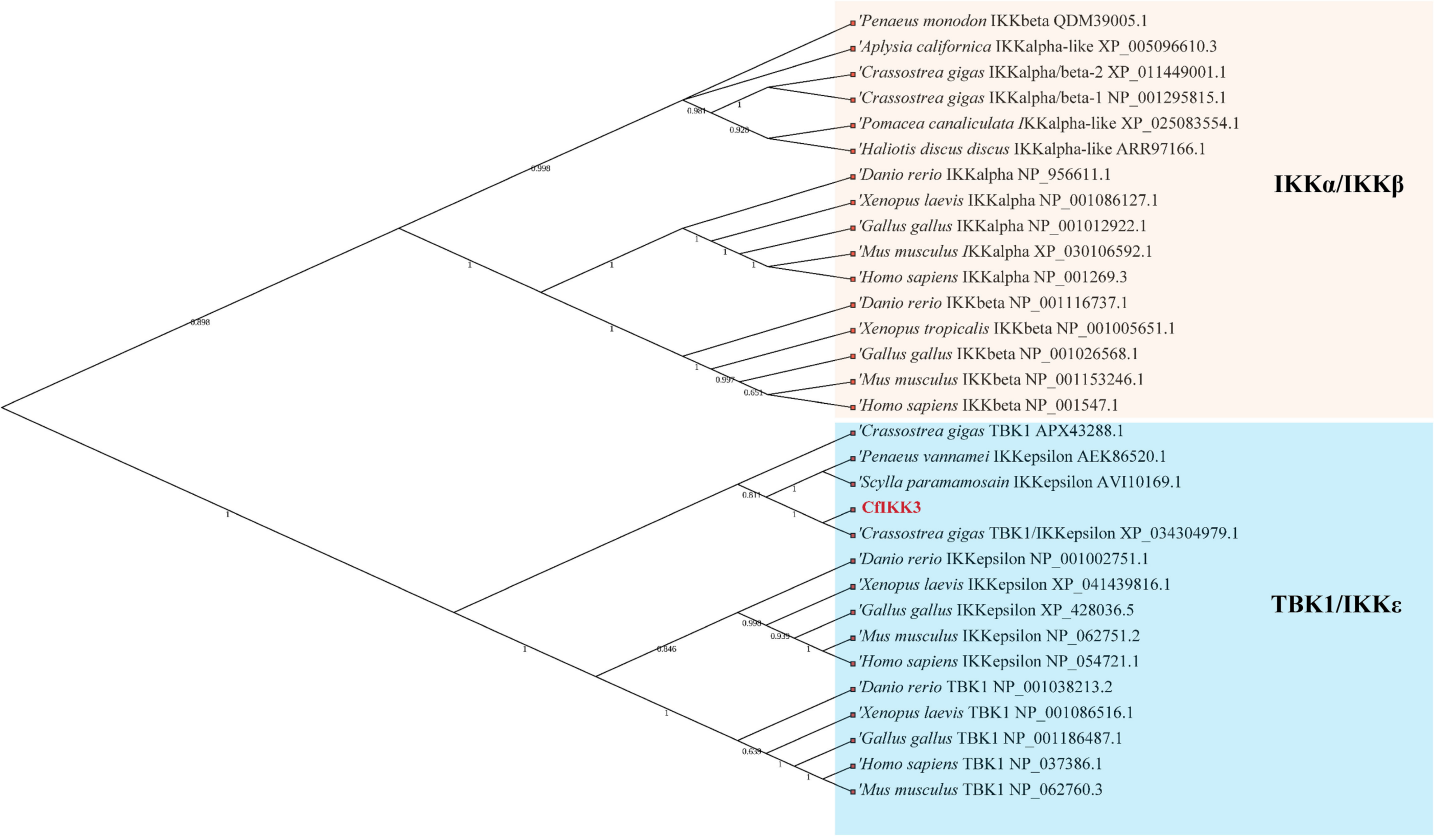
Phylogenetic tree of canonical IKKs and IKK-related kinases from various species. The phylogenetic tree was constructed using the neighbor-joining method with 1000 bootstrap replications. The tree is divided into two main branches: IKKα/IKKβ family members and TBK1/IKKϵ family members. The GenBank accession numbers of the protein sequences are also shown.

The CfIKK3 protein has a similar domain composition to vertebrate TBK1/IKKϵ family proteins, all of which have a kinase domain (KD), ULD, and CCD1 (**Figure 2A**). Multiple sequence alignment of the KD sequences in these proteins showed that this domain is highly conserved (**Figure 2B**). As a characteristic domain of the IKK protein, we speculated that this scallop IKK protein has a conserved function. Based on the results of the sequence alignment, we constructed a phylogenetic tree of the IKK proteins of different species (**Figure 3**). The figure clearly shows a bifurcation of IKK proteins into the IKKα/IKKβ family and the TBK1/IKKϵ family. Interestingly, CfIKK3 belongs to the TBK1/IKKϵ family and was first grouped with the TBK1/IKKϵ protein of other invertebrates, such as the Pacific oysters *Crassostea gigas* and *Litopenaeus vannamei*.

### 3.3 *CfIKK3* expression patterns

**Figure 4:**
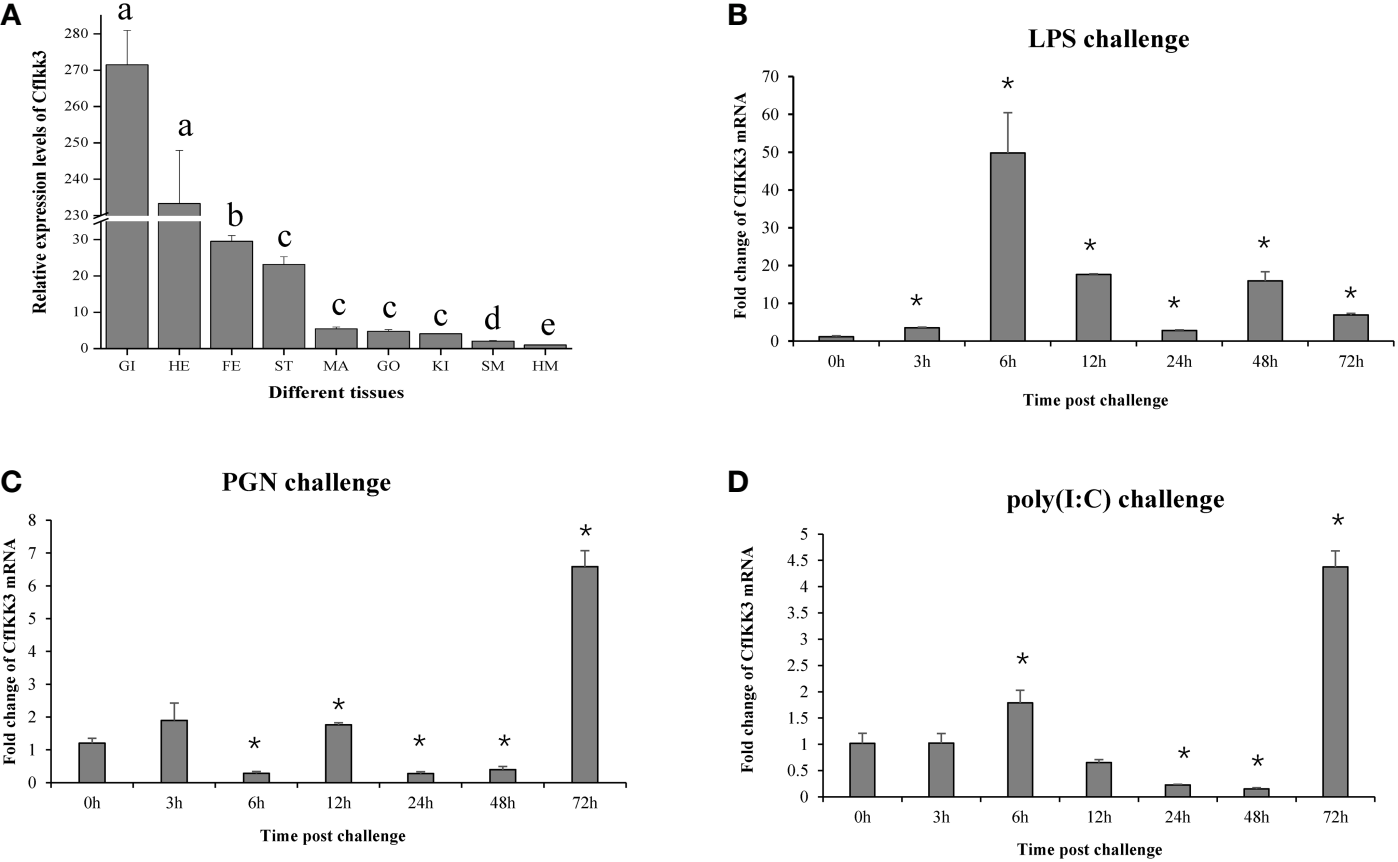
Expression profiles of *CfIKK3* mRNA in different tissues **(A)** and after LPS, PGN, and poly(I:C) challenges **(B–D)**, as determined using qRT-PCR. The *EF-1α* gene was chosen as the internal control gene, and hemocytes were used as a reference sample. The tissue-specific expression data were statistically analyzed with one-way ANOVA. For PAMP challenge assays, sampling was conducted at 0, 3, 6, 12, 24, 48, and 72 h after challenge. *EF-1α* gene was employed as the internal control, and time 0 h was considered as the reference sample. Vertical bars represent the mean ± SD (N = 3). *p < 0.05, according to the unpaired two-tailed *t*-test. GI, gill; HE, hepatopancreas; FE, feet; ST, striated muscle; MA, mantle; GO, gonad; KI, kidney; SM, smooth muscle; HM, hemocytes. LPS, lipopolysaccharide; PGN, peptidoglycan; poly(I:C), polyinosinic–polycytidylic acid.

The expression pattern of *CfIKK3* was verified by qRT-PCR. Tissue-specific expression of this gene is shown in **Figure 4A**. The CfIKK3 gene was detected in all tested scallop tissues, with the highest expression level in gill tissues. The expression level of this gene in blood cells was relatively lower. Simultaneously, to reveal the function of CfIKK3 in host innate immunity, we studied the temporal expression patterns of *CfIKK3* under bacterial or virus-associated PAMPs attack. qRT-PCR results showed that *CfIKK3* mRNA expression levels showed a significant inducible expression pattern after stimulation with bacterial-associated PAMPs (LPS, PGN) and virus-associated PAMP (poly(I:C)) (**Figures 4B–D**). For example, after LPS challenge, the expression of the CfIKK3 gene was significantly upregulated at 6 h after stimulation; after PGN challenge, the expression of the CfIKK3 gene was significantly increased at 72 h after stimulation; and after poly(I:C) challenge, the expression of the CfIKK3 gene was elevated at 6 h and 72 h after stimulation.

### 3.4 CfIKK3 interacts with CfMyD88 directly

**Figure 5:**
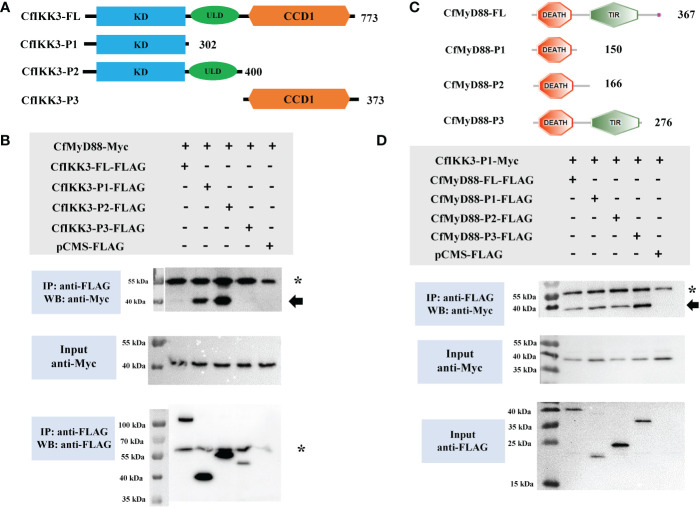
**(A)** Schematic representations of the wild-type full-length CfIKK3 protein (CfIKK3-FL) and truncated mutant CfIKK3-P1, CfIKK3-P2, and CfIKK3-P3 proteins. The protein lengths and domains are annotated. **(B)** Interaction between CfIKK3 and CfMyD88, as verified using co-immunoprecipitation (co-IP) assays. The co-IP of CfMyD88 proteins with various CfIKK3-FLAG proteins (CfIKK3-FL, CfIKK3-P1, CfIKK3-P2, and CfIKK3-P3) was facilitated using anti-FLAG M2 magnetic beads and analyzed by western blot assay using the anti-Myc antibody (top). Input samples were detected using anti-Myc (middle) and anti-FLAG antibodies (bottom), respectively, to confirm the Myc- and FLAG-fused protein expression. CfMyD88-Myc could be detected in the anti-FLAG immunoprecipitates of co-transfected (CfIKK3-FL, CfIKK3-P1, and CfIKK3-P2) cell extracts, indicating the interaction of CfMyD88-Myc with CfIKK3-FL-FLAG (weak interaction), CfIKK3-P1-FLAG, and CfIKK3-P2-FLAG. The asterisk represents the heavy chain of mouse IgG. **(C)** Schematic representations of the wild-type full-length CfMyD88 protein (CfMyD88-FL) and truncated mutant CfMyD88-P1, CfMyD88-P2, and CfMyD88-P3 proteins. The protein lengths and domains are annotated. **(D)** Interaction between CfIKK3-P1 and CfMyD88, as verified using co-immunoprecipitation (co-IP) assays. Co-IP results indicated the interaction of CfIKK3-P1-Myc with CfMyD88-FL-FLAG, CfMyD88-P1-FLAG, CfMyD88-P2-FLAG and CfMyD88-P3-FLAG. The asterisk represents the heavy chain of mouse IgG.

TLR family proteins are a key class of PPRs that play key roles in cellular innate immunity against bacteria and viruses. TLRs can recruit the adaptor protein MyD88 for immune signaling (27, 28). In the present study, we investigated the potential relationship between CfIKK3 and the adaptor protein MyD88 (GenBank accession number DQ249918.1). Notably, co-IP and immunoblotting results showed that CfIKK3 can directly interact with CfMyD88 (**Figure 5**). Additionally, truncated mutants of protein expression plasmids were constructed to explore the key protein domains that mediate the interaction of these two proteins (**Figures 5A, C**). As shown in **Figure 5B**, in addition to the wild-type CfIKK3, the CfIKK3-P1 mutant (KD only) and the CfIKK3-P2 mutant (KD + ULD) could also combine with CfMyD88, suggesting a critical role for the KD domain of CfIKK3 in mediating the interaction. Based on this result, we further investigated the binding of truncated CfMyD88 protein to the KD of CfIKK3. As shown in **Figure 5D**, in addition to the wild-type CfMyD88, the CfMyD88-P1 mutant (DEATH domain only), CfMyD88-P2 mutant (DEATH domain, the peptide length is slightly longer than that of CfMyD88-P1), and CfMyD88-P3 mutant (DEATH domain + TIR domain) could also interact with CfIKK3-P1, suggesting a critical role for the DEATH domain of CfMyD88 in mediating this interaction.

### 3.5 CfIKK3 interacts directly with CfMAVS

**Figure 6:**
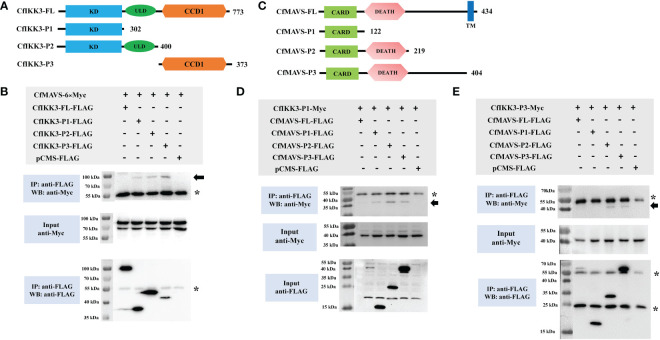
**(A)** Schematic representations of the wild-type full-length CfIKK3 protein (CfIKK3-FL) and truncated mutant CfIKK3-P1, CfIKK3-P2, and CfIKK3-P3 proteins. The protein lengths and domains are annotated. **(B)** Interaction between CfIKK3 and CfMAVS, as verified using co-immunoprecipitation (co-IP) assays. Co-IP results indicated the interaction of CfMAVS-6×Myc with CfIKK3-P1-FLAG, CfIKK3-P2-FLAG, and CfIKK3-P3-FLAG. Asterisk represents the heavy chain of mouse IgG. **(C)** Schematic representations of the wild-type full-length CfMAVS protein (CfMAVS-FL) and truncated mutant CfMAVS-P1, CfMAVS-P2, and CfMAVS-P3 proteins. The protein lengths and domains are annotated. **(D)** Interaction between CfIKK3-P1 and CfMAVS, as verified using co-immunoprecipitation (co-IP) assays. Co-IP results indicated the interaction of CfIKK3-P1-Myc with CfMAVS-P1-FLAG, CfMAVS-P2-FLAG, and CfMAVS-P3-FLAG. Asterisk represents the heavy chain of mouse IgG. **(E)** Interaction between CfIKK3-P3 and CfMAVS, as verified using co-immunoprecipitation (co-IP) assays. Co-IP results indicated the interaction of CfIKK3-P3-Myc with CfMAVS-P2-FLAG and CfMAVS-P3-FLAG. Asterisk represents the heavy chain or the light chain of mouse IgG.

In addition to TLR family proteins, RLR family proteins constitute another class of key PPRs (29, 30). RLR proteins are usually localized in the cytoplasm and transduce signals by recruiting adaptor protein MAVS (31, 32). We investigated the relationship between CfIKK3 and the adaptor protein MAVS (GenBank accession number OM069740, **Figure 6**). Co-ip and western blotting results did not indicate an interaction between the full-length CfIKK3 and CfMAVS. However, CfIKK3-P1, CfIKK3-P2, and CfIKK3-P3 (CCD1 only) could directly bind to CfMAVS, suggesting that both KD and CCD1 of CfIKK3 may be involved in the interaction with MAVS (**Figure 6B**). Furthermore, we constructed a truncated CfMAVS (**Figure 6C**) and studied the interaction of each truncated MAVS protein with CfIKK3-P1. The results showed that CfMAVS-P1 (CARD only), CfMAVS-P2 (CARD + DEATH domain), and CfMAVS-P3 (full-length CfMAVS without TM domain) could directly bind to CfIKK3-P1, suggesting that the CARD of CfMAVS might be sufficient for binding to the KD of CfIKK3 (**Figure 6D**). At the same time, we also studied the interaction of each truncate protein of CfMAVS with CfIKK3-P3. The results showed that both CfMAVS-P2 and P3 could directly bind to CfIKK3-P3, while CfMAVS-P1 did not, implying that the DEATH domain of CfMAVS mediates binding to the CCD1 domain of CfIKK3 (**Figure 6E**).

### 3.6 CfIKK3 could form homodimers and bind CfIKK2

**Figure 7:**
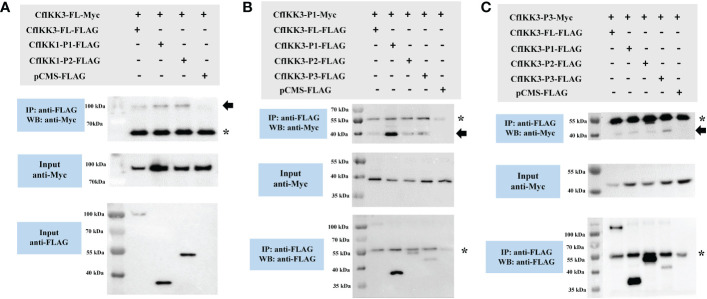
Co-IP assays showed that CfIKK3 can form homodimers. **(A)** The self-association of CfIKK3 was verified using co-immunoprecipitation (co-IP) assays. Co-IP results indicated the interaction of CfIKK3-FL-Myc with CfIKK3-FL-FLAG, CfIKK3-P1-FLAG and CfIKK3-P2-FLAG. **(B)** Interaction between the CfIKK3 kinase domain (CfIKK3-P1-Myc) and various CfIKK3-FLAG proteins (CfIKK3-FL, CfIKK3-P1, CfIKK3-P2, and CfIKK3-P3), as verified using co-immunoprecipitation assays. The results showed that CfIKK3-P1-Myc could interact with CfIKK3-FL-FLAG, CfIKK3-P1-FLAG, CfIKK3-P2-FLAG, and CfIKK3-P3-flag. **(C)** Interaction between the CfIKK3 CCD1 domain (CfIKK3-P3-Myc) and various CfIKK3-FLAG proteins (CfIKK3-FL, CfIKK3-P1, CfIKK3-P2, and CfIKK3-P3), as verified using co-immunoprecipitation assays. The results showed that CfIKK3-P3-Myc could interact with CfIKK3-FL-FLAG, CfIKK3-P1-FLAG, CfIKK3-P2-FLAG, and CfIKK3-P3-flag. Asterisk represents the heavy chain of mouse IgG.

In this study, co-IP assays were conducted to determine the binding relationship between CfIKK3 and our previously cloned IKK family proteins (CfIKK1 and CfIKK2). The results showed that CfIKK3 did not substantially bind to CfIKK1 (results not shown), but CfIKK3 had obvious self-association (**Figure 7A**). Furthermore, the regions mediating homodimer formation were verified using constructed truncations. Interestingly, the results showed that CfIKK3-FL-flag, CfIKK3-P1-flag, CfIKK3-P2-flag, and CfIKK3-P3-flag interacted with CfIKK3-P1-myc (KD only) (**Figure 7B**). The results also showed that CfIKK3-FL-flag, CfIKK3-P1-flag, CfIKK3-P2-flag, and CfIKK3-P3-flag interacted with CfIKK3-P3-myc (**Figure 7C**).

**Figure 8:**
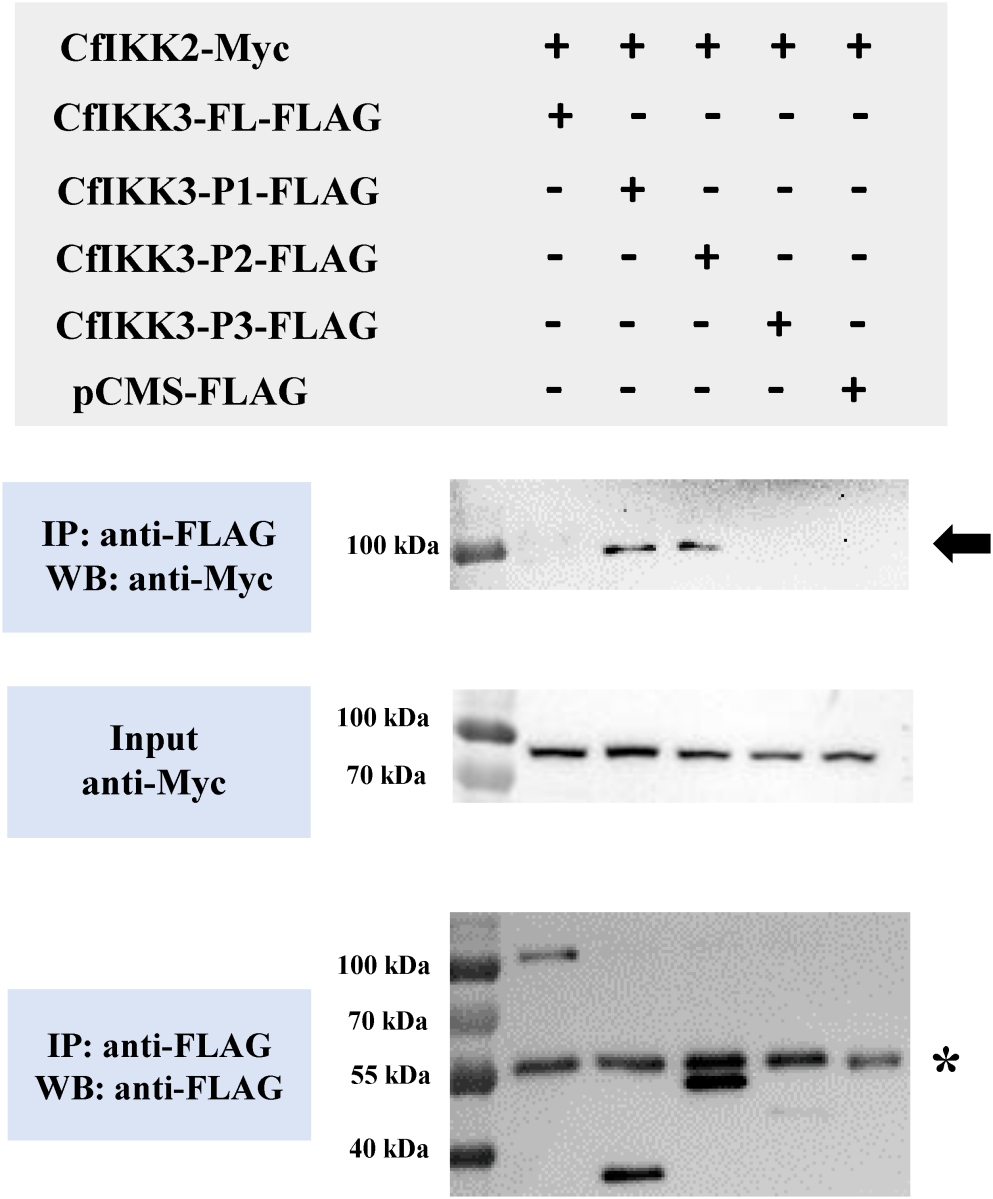
Co-immunoprecipitation (Co-IP) assays showed that TBK1/IKKϵ family members of Zhikong scallop (CfIKK2 and CfIKK3) could bind each other. Co-IP results indicated the interaction of CfIKK2-Myc with CfIKK3-P1-FLAG and CfIKK3-P2-FLAG. Asterisk represents the heavy chain of mouse IgG.

In addition, co-ip results showed that CfIKK3 might be interacting with CfIKK2, another TBK1/IKKϵ family member of *C. farreri*. CfIKK3-P1-flag and CfIKK3-P2-flag bound to CfIKK2-myc, suggesting that the KD of CfIKK3 might be sufficient for binding to CfIKK2 (**Figure 8**).

### 3.7 CfIKK3 overexpression activates NF-κB reporter gene

**Figure 9:**
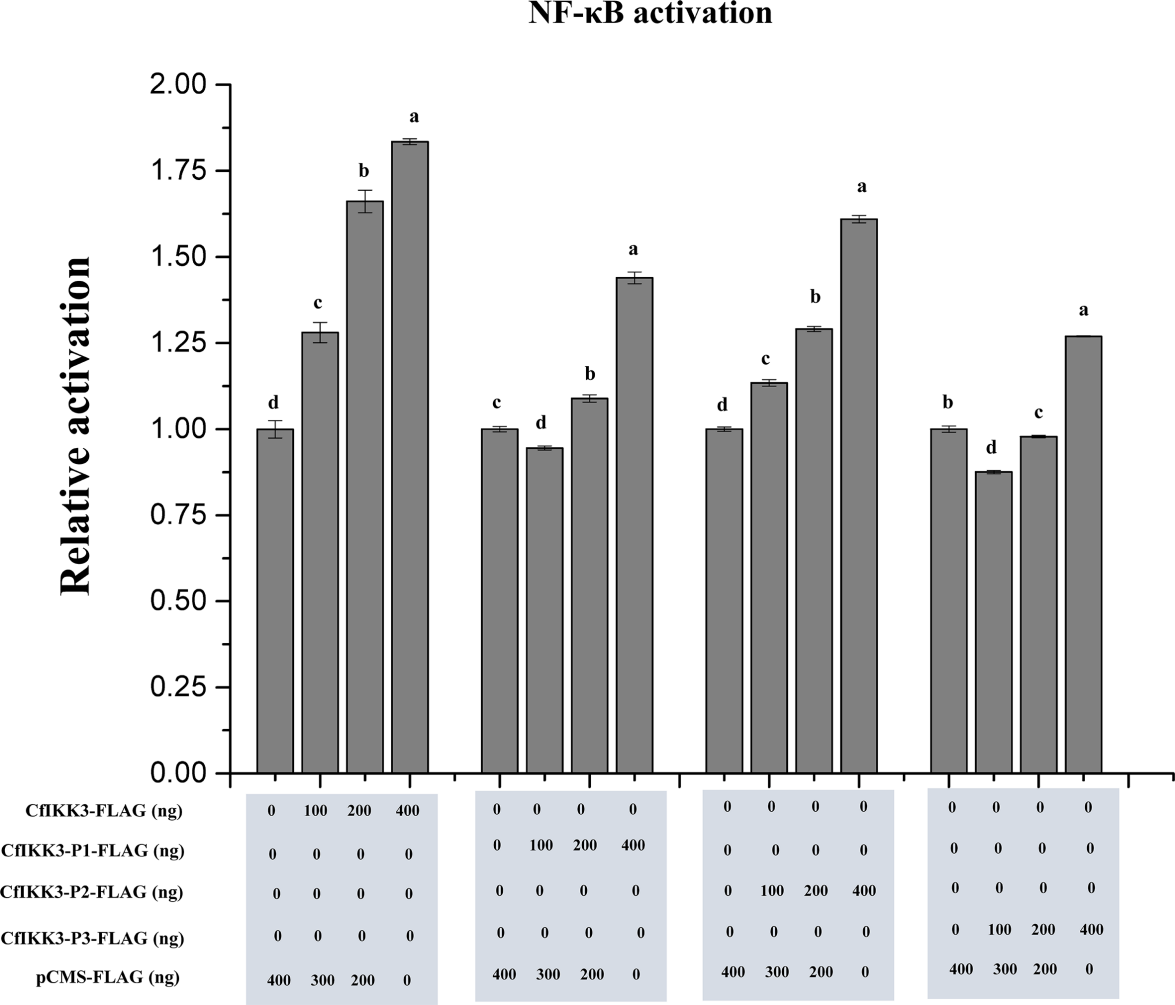
Activation of *NF-κB* reporter gene in HEK293T cells by overexpression of CfIKK3 protein or its truncated mutants, as verified using dual luciferase reporter gene assays. The results are represented as fold changes in the experimental groups compared to those in the control groups. Vertical bars represent mean ± SD (n = 3). Different letters indicate significant differences (*p* < 0.05).

DLR gene assays were employed to detect the possible activation of CfIKK3 on target reporter genes (NF-κB or ISRE). The results showed that the overexpression of CfIKK3 in HEK293T cells did not activate the ISRE reporter gene (data not shown). However, overexpression of CfIKK3 can significantly activate the NF-κB signaling pathway, and this activation has a significant dose-dependent effect. From the results of the activation of NF-κB by each truncated mutant protein, KD + ULD appeared to have a significant dose-dependent activation effect on NF-κB reporter genes (**Figure 9**).

## 4 Discussion

Innate immunity is a key defense mechanism in animals, especially invertebrates, to evade pathogenic microorganisms in the environment. The role of innate immunity is more important in invertebrates, because a perfect adaptive immune system is not yet evolved. IKK is a key signaling molecule in animal innate immune pathways that usually serves as a convergence point for many signal transduction pathways and plays a key role in activating key downstream transcription factors. In this study, we selected the Zhikong scallop, a species of ecological and evolutionary importance, as the research object. One of the IKK family genes (*CfIKK3*) was cloned and its immune function was studied. These results can contribute to the analysis of the innate immune mechanisms of shellfish and the development of comparative immunology.

Using genome prediction and PCR amplification, we successfully cloned the complete coding sequence of *CfIKK3*, which encodes a polypeptide of 773 amino acids. The CfIKK3 protein shares a similar domain architecture to that of the vertebral TBK1/IKKϵ proteins. All these TBK1/IKKϵ family proteins contain KD, ULD, and CCD1. At the same time, sequence alignment of the KDs of different species showed that the protein sequence of this domain was highly conserved, suggesting conservation of the function of the CfIKK3 protein. During phylogenetic analysis, CfIKK3 and other invertebrate TBK1/IKKϵ proteins clustered into a branch, indicating that CfIKK3 belongs to invertebrate TBK1/IKKϵ family. However, we noticed that the TBK1 and IKKϵ family proteins of vertebrates were first clustered together, suggesting that the differentiation of TBK1 and IKKϵ might have occured after the emergence of vertebrates. Therefore, for the scallop CfIKK3, we can only speculate that it belongs to the invertebrate TBK1/IKKϵ family, but not further distinguish whether the protein is a TBK1 protein or an IKKϵ protein.

Next, we investigated the expression pattern of *CfIKK3*. *CfIKK3* mRNA was expressed in all tested scallop tissues and was highly expressed in gill tissues, which may be due to the frequent exposure of gill tissues of filter-fed scallops to external pathogens. Subsequently, we carried out stimulation experiments with bacteria, viruses, and related PAMPs. In previous studies, the expression of the scallop IKK1 gene was induced by LPS, PGN, and poly(I:C) stimuli (19), in this research, the results showed that the expression of *CfIKK3* mRNA was significantly induced after stimulation with bacteria, viruses, and related PAMPs, indicating that this gene plays an important role in the innate immunity of scallops against bacteria and viruses. At the same time, we noted that changes in the response time and expression level of *CfIKK3* mRNA were also different after different PAMPs stimuli, suggesting that CfIKK3 plays different roles in response to different stimuli or responds by participating in different immune pathways.

In mammals and other higher animals, TBK1/IKKϵ protein plays a key role in the process of undertaking upstream immune signals and activating downstream transcription factors. According to previous reports, Zhikong scallop appears to have functional TLR and RLR signaling pathways. Therefore, we wondered whether CfIKK3 is involved in the signal transduction of the scallop TLR and RLR innate immune pathways. Therefore, we selected the key adaptor proteins MyD88 and MAVS in the TLR and RLR pathways and verified the interaction between CfIKK3 and adaptor proteins by co-IP experiments. First, the co-IP results showed that CfIKK3 clearly interacted with CfMyD88. Through further truncation construction and interaction experiments, we confirmed that the KD of CfIKK3 and the DEATH domain of CfMyD88 were sufficient to mediate the interaction of these two proteins. Simultaneously, the co-IP results showed that CfIKK3 could also directly bind to CfMAVS. Further experimental results showed that the binding of CfIKK3 and CfMAVS was complicated, and both KD and CCD1 of CfIKK3 could bind CfMAVS. Furthermore, by constructing a truncation of CfMAVS, we found that the CARD of CfMAVS was sufficient for binding to the KD of CfIKK3, whereas the DEATH domain mediates binding of CfMAVS to the CCD1 of CfIKK3. During the experiment, the full-length forms of CfIKK3 and CfMAVS proteins appeared to bind poorly to other proteins. It is speculated that there are certain regulatory motifs in the full-length protein that play a key role in regulating protein activity. Taken together, our study further confirmed the existence of TLR and RLR signaling pathways in scallops and demonstrated that CfIKK3 plays a key role in mediating TLR and RLR signaling pathways.

Mammalian IKK proteins typically dimerize to form IKK complexes, which are important for signal transduction and target protein activation (6). In the current study, the results of co-IP assays confirmed the self-association of the CfIKK3 protein and its interaction with CfIKK2, which may play a key role in the activation of downstream target proteins. At the same time, the interaction experiments of the truncated mutants revealed the key roles of the domains KD and CCD1 in the homodimerization of CfIKK3. The participation of multiple domains may ensure the structural stability of IKK complexes formed by CfIKK3 for proper functioning.

CfIKK3 is involved in RLR and TLR signaling pathways and also forms IKK complexes. However, little is known about the downstream regulatory genes of IKK3 in scallops. Therefore, in this study, we verified the activation effect of CfIKK3 on NF-κB, a key gene in innate immunity, using DLR experiments. The results of DLR assays showed that overexpression of CfIKK3 in HEK293T cells did not activate the ISRE reporter gene, but could significantly activate the NF-κB signaling pathway. Based on this, we speculated that after receiving upstream immune signals and activation, CfIKK3 may exert its immune function mainly by activating NF-κB, a key transcription factor in innate immunity. The regulation of CfIKK3 and the activation mechanism of NF-κB require further detailed research.

**Figure 10:**
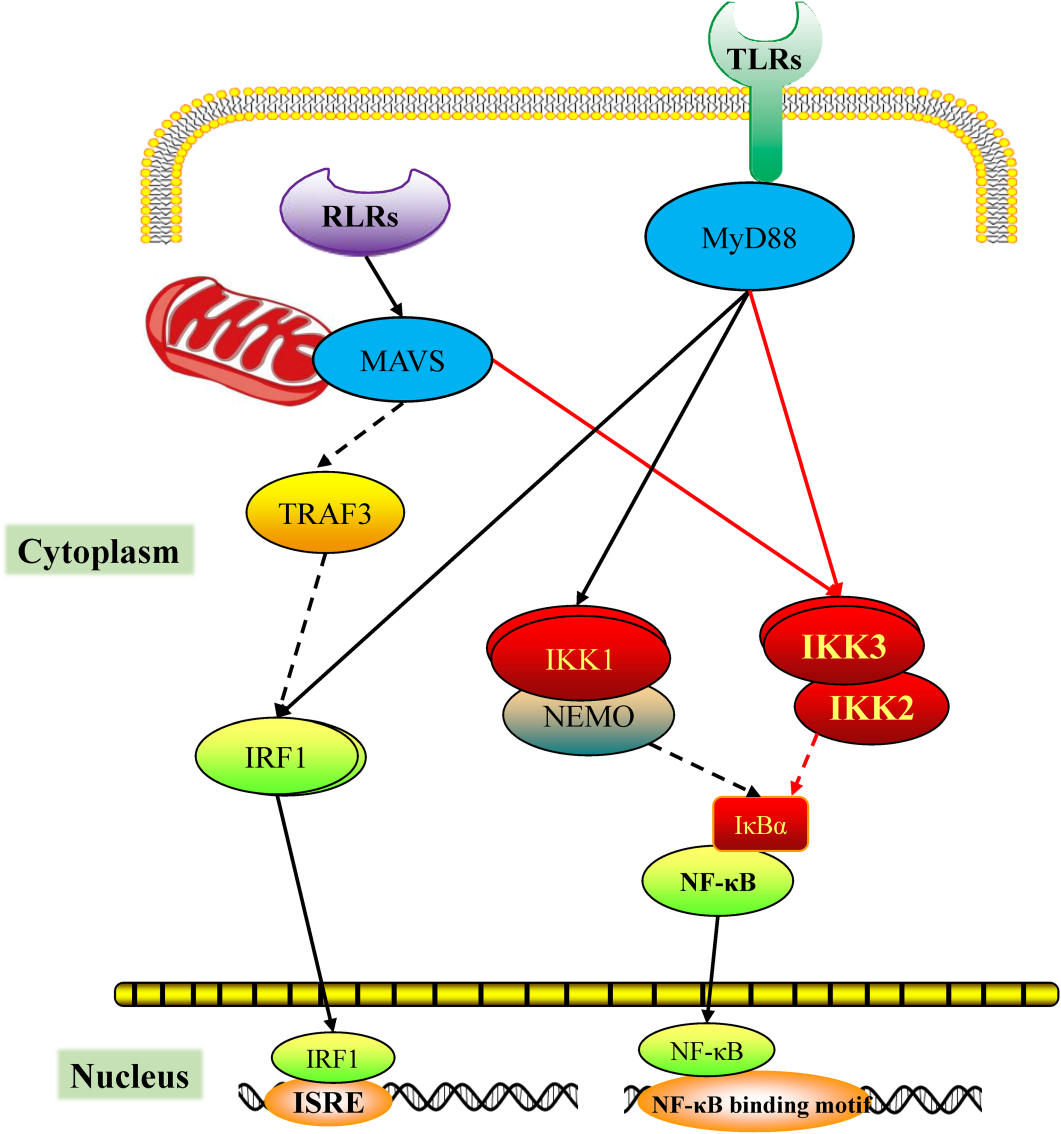
Predicted diagram of the scallop the RIG-I-like receptor (RLR) and toll-like receptor (TLR) signaling mediated by CfIKK3. The PAMPs challenge activates the scallop pattern recognition receptors RLR and TLR, which bind the corresponding adaptor molecules CfMAVS or CfMyD88, respectively. Next, CfMAVS and CfMyD88 recruit CfIKK3 for signaling. The dimerization of CfIKK3 and the CfIKK2-CfIKK3 interaction may be crucial steps of IKK complex formation. The IKK complex activates the downstream nuclear factor-κB (NF-κB).

Based on the results from the current and previous studies on the TLR and RLR pathways of scallops, we outlined the role of CfIKK3 in TLR and RLR signaling transduction pathways (**Figure 10**). Briefly, the invasion of pathogenic microorganisms activates PRRs such as scallop TLRs and RLRs, which interact with their adaptor proteins CfMyD88 and CfMAVS, respectively. The adaptor proteins recruit CfIKK3, which subsequently dimerizes and binds to CfIKK2 to form the IKK complex. The IKK complex further activates downstream transcription factors such as NF-κB to initiate a cellular immune response. However, further studies are required to elucidate the specific details of signal transduction and transcription factor activation.

In conclusion, a TBK1/IKKϵ family gene of invertebrates was identified in the Zhikong scallop (*C. farreri*) and was termed *CfIKK3*. *CfIKK3* mRNA was distributed in all scallop tissues and its expression was significantly induced by LPS, PGN, and poly(I:C) challenge. CfIKK3 could bind to CfMAVS and CfMyD88, indicating its involvement in scallop RLR and TLR signaling. Moreover, the self-association of CfIKK3 and its interaction with CfIKK2 may be a crucial step in self-activation as well as activation of downstream NF-κB. Taken together, our results reveal that CfIKK3 is involved in the signal transduction of the TLR and RLR pathways in scallops and participates in the regulation of innate immunity. The results of our study are conducive to further in-depth analysis of the innate immune mechanism of scallops and lay a theoretical foundation to aid healthy breeding of scallops and the development of comparative immunology studies.

## Data availability statement

The datasets presented in this study can be found in online repositories. The names of the repository/repositories and accession number(s) can be found below: https://www.ncbi.nlm.nih.gov/genbank/, OP764589.

## Ethics statement

The studies involving animals were reviewed and approved by the respective Animal Research and Ethics Committees of Ludong University.

## Author contributions

BH and XTW conceived and designed the study. WL, JM, JC, FCL, LL, NF, FSL, YZ, XZ, XNW, and XMW performed experiments. LW, YL, MZ, and YH analyzed the data. BH, WL, and XTW wrote the manuscript. All authors have read and approved the final manuscript.
